# Unpacking the dual-path trust mechanism driving students' continuance intention toward campus GenAI: a hybrid SEM-machine learning approach within the S-O-R framework

**DOI:** 10.3389/fpsyg.2026.1903629

**Published:** 2026-07-17

**Authors:** Bingyao Li, Tianzuo Yu

**Affiliations:** 1 Student Affairs Office, Shanghai Business School, Shanghai, China; 2 School of Education, Shanghai Jiao Tong University, Shanghai, China

**Keywords:** continuance intention (CUI), dual-path trust, generative AI, higher education, S-O-R framework, structural equation modeling, machine learning

## Abstract

As generative artificial intelligence agents become embedded in higher education, understanding students' continuance intention (CUI) is critical. To address the underexplored mechanisms regarding how campus AI environments are associated with user trust, this study adopts the Stimulus-Organism-Response (S-O-R) framework to investigate a dual-path trust mechanism: AI System-like Trust (AST) and AI Human-like Trust (AHT). Through a hybrid methodology combining Partial Least Squares Structural Equation Modeling (PLS-SEM) and non-parametric Random Forest classification, this study analyzes university students' CUI toward campus GenAI. The findings demonstrate that Information Quality (IQ) and Service Quality (SQ) are positively associated with both AST and AHT pathways. Conversely, Facilitating Conditions (FC) and Performance Expectancy (PE) do not exhibit independent linear associations within the simultaneous structural estimation. Rather than indicating an empirical absence of functional value, this configuration uncovers a holistic evaluation mechanism among digital natives, where basic functional affordances operate as baseline hygiene constraints whose variance is subsumed by quality attributes. Notably, while both AST and AHT exert concurrently significant linear driving forces on CUI within the structural path framework, the exploratory machine learning analysis clarifies that under recursive partitioning, AHT exhibits a higher relative predictive weight. Furthermore, a localized asymmetry operates within the linear mediation channels, where SQ selectively mobilizes AHT over AST to drive continuous usage. *Post-hoc* inspections imply that PE operates through a non-linear threshold dynamic, transitioning into an active predictive catalyst primarily within the positive spectrum of user perception. Our study contributes to campus AI services evaluation by highlighting that within the contemporary GenAI ecosystem, students' evaluative focus prioritizes relational and epistemic quality over functional accessibility. Campus application administrators and developers are advised to prioritize content rigor and responsive, empathetic interaction design to cultivate the relational trust relevant for sustained engagement.

## 1 Introduction

The rapid proliferation of generative artificial intelligence (GenAI) has catalyzed a fundamental shift in higher education, with AI-powered conversational agents emerging as integral tools for campus management and student support services (Bernabei et al., 2023). In China, institutions are increasingly deploying these systems to streamline administrative workflows and enhance the academic experience for digitally proficient learners (Ma and Huo, 2023). Yet the long-term value of such deployments depends not on initial uptake but on students' continuance intention (CUI)—the sustained behavioral commitment to engage with the technology beyond the exploratory phase (Sohail et al., 2023; Eagan et al., 2024). As campus AI agents become institutionally embedded, understanding what drives this post-adoption persistence has become a pressing concern for both researchers and practitioners (Maheshwari, 2024).

A growing body of evidence identifies trust as the central psychological mechanism governing sustained AI engagement (Glikson and Woolley, 2020). Within educational contexts, scholars have examined how AI literacy, social norms, and perceived competence shape students' willingness to rely on AI systems (Wang et al., 2025; Das and Chernova, 2020). Critically, recent work has moved beyond treating trust as a unidimensional construct. Building upon the foundational dual-dimensional trust structures established by Lankton et al. (2015)—AI system-like trust (AST), which captures cognitive assessments of technical reliability, and AI human-like trust (AHT), which captures affective attributions of social benevolence, scholars have increasingly examined dual trust pathways across various technology-mediated contexts. These include general AI acceptance frameworks (e.g., Choung et al., 2023) as well as platform environments characterized by distinct technical and social entities (e.g., Califf et al., 2020). Prior empirical efforts have predominantly modeled AST and AHT as static, parallel antecedents within expanded, direct-effect acceptance frameworks, thereby leaving the dynamic activation mechanisms under-specified. Specifically, they fail to explain how heterogeneous environmental configurations differentially trigger these cognitive vs. affective trust states.

This study addresses this gap by synthesizing the Unified Theory of Acceptance and Use of Technology (UTAUT) and the Information Systems Success (ISS) model within the overarching Stimulus-Organism-Response (S-O-R) paradigm. By positioning the dual-path trust mechanism as parallel, dynamic mediating organisms (O), we structurally map how multi-dimensional campus AI environmental stimuli (S) systematically activate distinct trust dimensions to govern post-adoption continuance (R). Specifically, we operationalize performance expectancy (PE) and facilitating conditions (FC) from the UTAUT model, alongside information quality (IQ) and service quality (SQ) from the ISS model, as the environmental stimuli (S). These psychological and system stimuli are hypothesized to differentially trigger students' AST and AHT (O), which ultimately drive their continuous usage intention (R) toward campus AI agents. Methodologically, the study employs a hybrid approach combining Partial Least Squares Structural Equation Modeling (PLS-SEM) with Random Forest classification, enabling both theory-based structural estimation and the capturing of distinct data-driven predictive patterns. Crucially, our findings reconcile a key empirical divergence between the linear structural pathways and the algorithmic predictive importance regarding performance expectancy, offering a more nuanced, methodologically inclusive account of how campus AI environments are associated with student engagement.

## 2 Literature review

### 2.1 Generative AI in higher education: from adoption to continuance

The proliferation of GenAI within higher education ecosystems has precipitated a critical transition in research focus: shifting from nascent adoption toward the complexities of sustained post-adoption behavior. While seminal models originally established that perceived utility and technical accessibility are the primary catalysts for initial uptake (Davis, 1989; Venkatesh et al., 2012), the institutionalization of these tools necessitates a deeper understanding of the mechanisms driving students' long-term behavioral persistence (Baig and Yadegaridehkordi, 2025). In this maturing landscape, Continuance Intention (CUI), defined as the psychological predisposition to sustain system usage beyond the exploratory phase, has emerged as a pivotal determinant of educational technology success (Bhattacherjee, 2001). Emerging evidence suggests that in AI-mediated environments, the transition from adoption to continuance is governed less by utilitarian calculations and more by the evolution of trust. Consequently, this study positions the dual-path trust as the central explanatory mechanism for CUI, investigating how specific environmental stimuli within the campus AI architecture relate to and support this psychological state.

### 2.2 Theoretical foundations of technology acceptance

#### 2.2.1 Technology acceptance model (TAM)

The Technology Acceptance Model (TAM), introduced by Davis (1989), remains a cornerstone of information systems (IS) research, positing perceived usefulness (PU) and perceived ease of use (PEOU) as the primary determinants of behavioral intention (Davis, 1989). While TAM effectively captures these generalized cognitive perceptions, it typically treats complex environmental and system characteristics as a broad, undifferentiated category of “external variables.” Consequently, the classical framework lacks a structured architecture to explain how distinct types of environmental stimuli (such as technical features, system, and information qualities) systematically activate unique configurations of internal trusting states. This parsimony becomes a significant limitation in the context of GenAI, where the diversity of environmental inputs cannot be adequately captured by monolithic external factors. To resolve this limitation, this study leverages the Stimulus-Organism-Response (S-O-R) framework. By structuring environmental inputs as explicit Stimuli, this framework offers a coherent approach to map unique technological attributes directly onto the Organism's dual-path trust mechanisms—a theoretical integration recently validated in empirical research on university students' interaction with campus generative AI tools (Li et al., 2026). Consequently, our model provides a more robust and comprehensive explanatory framework for students' continuance intention.

#### 2.2.2 Unified theory of acceptance and use of technology (UTAUT)

(Venkatesh et al. 2003) synthesized diverse acceptance models into the Unified Theory of Acceptance and Use of Technology (UTAUT), identifying performance expectancy (PE), effort expectancy (EE), social influence (SI), and facilitating conditions (FC) as the core drivers of intent. While UTAUT has been extensively applied to analyze initial GenAI adoption in higher education, frequently highlighting PE and FC as significant predictors (Rahi et al., 2018), it typically assumes a direct-effect pathway between these constructs and behavior. This study refines this approach by operationalizing PE and FC as stimuli within an S-O-R architecture, thereby exploring how these functional affordances are filtered through the student's internal organism, specifically their trust, before manifesting as a commitment to continued use.

#### 2.2.3 Information Systems Success (ISS) model

DeLone and McLean's (2003) updated IS Success model identifies information quality (IQ), system quality, and service quality (SQ) as the three primary dimensions influencing user satisfaction and net benefits. The model has been extensively validated in e-commerce, healthcare IS, and more recently in AI-powered service platforms (Veeramootoo et al., 2018). A recent conceptual framework for AI-enabled service systems specifically highlights reliability, responsiveness, and relational quality as key determinants of user trust and sustained engagement (Colgate and Colgate, 2026). By incorporating IQ and SQ as stimulus variables, this study complements the functional focus of UTAUT with the output-oriented focus of the ISS model, providing a multi-dimensional representation of the campus AI environment.

### 2.3 The Stimulus-Organism-Response (S-O-R) framework

The S-O-R framework, originating in Mehrabian and Russell's environmental psychology work, proposes that environmental stimuli (S) elicit internal psychological states in the organism (O), which in turn drive observable behavioral responses (R) (Mehrabian and Russell, 1974). In IS research, the framework has been productively applied to explain user behavior in e-commerce, social media, and smart technology contexts (Ding and Lee, 2024; Liu et al., 2025; Chen X. et al., 2025), where it offers a structural advantage over TAM and UTAUT: it explicitly models the mediating role of internal psychological states rather than treating them as direct antecedents of behavior (Wang and Long, 2025). Recent applications in AI-enhanced educational settings have demonstrated the framework's capacity to capture how features of the digital learning environment, including system quality, interactivity, and social presence, relate to cognitive and affective states that correlate with engagement and continuance (Zhai et al., 2023).

The S-O-R framework is particularly well-suited to the present study for two reasons. First, it accommodates multiple, heterogeneous stimuli drawn from different theoretical traditions (UTAUT and ISS) within a single coherent architecture. Second, it provides a principled basis for modeling dual-path mediation: the organism layer can represent both cognitive (system-like) and affective (human-like) trust simultaneously, capturing the concurrent and multi-dimensional activation of both cognitive and affective trust, allowing for a comparative analysis of how environmental inputs translate into behavioral commitment through distinct psychological pathways. A recent study applying the S-O-R framework to GenAI tools in higher education confirmed that this architecture effectively captures the trust-mediated pathway from facilitating conditions and performance expectancy to usage intention (Zhang et al., 2025).

### 2.4 Trust in human-AI interaction

#### 2.4.1 Conceptualizations of trust in IS research

Trust has been theorized in IS research along two primary dimensions. McAllister distinguished cognition-based trust—grounded in rational assessments of competence, reliability, and track record—from affect-based trust, which develops through emotional bonds and perceived benevolence (McAllister, 1995). This distinction has remained influential across decades of IS research and has been extended to technology contexts, where users evaluate systems through both functional and relational lenses (Glikson and Woolley, 2020). Mayer et al.'s ability-benevolence-integrity framework further decomposed trust into assessable components that map naturally onto AI system evaluation: ability corresponds to technical competence, benevolence to perceived care for user interests, and integrity to consistency and ethical alignment (Mayer et al., 1995).

#### 2.4.2 Dual-dimensional trust in AI systems

The application of dual-dimensional trust to AI systems has gained significant traction in recent years. Lankton et al. (2015) argued that users evaluate technology platforms through two distinct trusting belief structures: system-like trust (AST), which reflects functional assessments of reliability, security, and performance consistency, and human-like trust (AHT), which captures social attributions of benevolence, integrity, and empathy toward the AI agent. Choung et al. (2023) provided empirical validation of this distinction within a TAM framework, demonstrating that both system-like and human-like trust significantly shape AI acceptance, with human-like trust playing a particularly critical role in fostering trusting intentions. More recently, research on generative AI has confirmed that cognitive trust in AI functionality serves as a “competence-to-affect” theoretical progression (Huynh and Aichner, 2025). While maintaining a parallel structural design, this study investigates the relative and complementary contributions of these dual trust dimensions to understand their respective pathways to post-adoption continuance usage without assuming an inherent hierarchical dominance of one over the other. Critically, the analytical framework is structured to evaluate whether technical rationality and emotional connection operate with symmetrical direct intensity, while simultaneously verifying the specific presence of localized asymmetries in how output and service-driven stimuli are channeled through these two psychological mechanisms.

#### 2.4.3 Trust as a mediator of continuance intention

The role of trust as a mediator between system output quality perceptions and continuance intention is well-established in IS research. Studies on chatbot services have consistently found that both cognitive and affective trust mediate the relationship between service quality and users' sustained engagement (Ashfaq et al., 2020; Balakrishnan et al., 2022). In the context of GenAI, human-like trust encompassing perceived empathy and ethical alignment operates alongside system-like trust to shape continuance intention, which suggests that relational dimensions of trust function as vital sustaining mechanisms as AI systems mature (Xia et al., 2026). This study builds on these empirical foundations by modeling both system-like trust and human-like trust as mediating organisms within the S-O-R architecture. The analytical framework is structured to evaluate whether technical rationality and emotional connection operate with symmetrical direct intensity, while simultaneously verifying the specific presence of localized asymmetries in how output and service-driven stimuli are channeled through these two psychological mechanisms to drive CUI.

### 2.5 Analytical methods: SEM and explainable machine learning

#### 2.5.1 Partial least squares structural equation modeling (PLS-SEM)

Structural Equation Modeling is the dominant analytical method in IS acceptance research, enabling simultaneous estimation of measurement and structural relationships among latent constructs (Leguina, 2015). The choice between covariance-based SEM (CB-SEM) and partial least squares SEM (PLS-SEM) depends on the research objective: CB-SEM is preferred when the goal is theory confirmation and model fit assessment, while PLS-SEM is better suited to exploratory and prediction-oriented research with complex mediation structures (Hair et al., 2019; Usakli and Rasoolimanesh, 2023). This study adopts PLS-SEM for three reasons. First, the research is prediction-oriented, seeking to explain variance in trust and continuance intention rather than to confirm a pre-specified covariance structure. Second, PLS-SEM imposes no distributional assumptions, making it robust to the non-normality commonly observed in Likert-scale survey data. Third, PLS-SEM is well-established in IS acceptance research for modeling complex mediation chains involving multiple latent constructs (Hair et al., 2019), which is precisely the dual-path trust mechanism examined here.

#### 2.5.2 Random forest classification

A recognized limitation of PLS-SEM is its assumption of linear relationships; it establishes theoretically grounded causal pathways but cannot capture non-linear interactions among predictors (Bao et al., 2026). Random Forest (RF), an ensemble learning method that constructs a large number of decision trees on bootstrapped subsamples and aggregates their predictions, addresses this limitation by providing model-agnostic feature importance rankings that are robust to non-linearity and variable interactions (Breiman, 2001).

#### 2.5.3 Rationale for the hybrid approach

The combination of SEM and Explainable Machine Learning (XML) in this study serves a dual purpose. SEM tests the theoretically derived causal pathways, specifically whether the dual-path trust mechanism mediates the stimulus-response relationship, and provides standardized path coefficients for hypothesis evaluation. XML then independently ranks all predictors by their contribution to CUI prediction, providing a data-driven validation of the SEM findings and revealing whether any non-significant SEM paths carry predictive weight that the linear model misses. This hybrid design strengthens both the explanatory and predictive validity of the study, addressing calls in the IS literature for methodological pluralism in technology acceptance research and yielding deeper insights into the factors driving sustained commitment to GenAI tools (Zeng et al., 2026).

## 3 Research model and hypothesis

Originating from environmental psychology, the Stimulus-Organism-Response (S-O-R) paradigm posits that environmental cues (S) systematically alter individuals' internal cognitive and affective states (O), which subsequently compel their behavioral outcomes (R) (Mehrabian and Russell, 1974). Given its robust applicability in capturing complex user-technology dynamics within technology-rich and AI-enhanced educational settings (Cao and Sun, 2018; Pan et al., 2024), the S-O-R framework serves as the theoretical anchor for this study. Within this architecture, stimuli comprise external technological or instructional affordances (Chen et al., 2022); the organism represents the mediating cognitive appraisals and emotional states that interpret these inputs (Jacoby, 2002); and responses denote observable behavioral trajectories, such as approach or avoidance intentions (Zhai et al., 2020).

To establish an analytical foundation that aligns with the multi-dimensional scope of this study, our methodological design is explicitly structured to support a comprehensive theoretical framework integrating the S-O-R, ISS, and UTAUT models. Rather than horizontally combining multiple frameworks, our approach employs a hierarchical integration, where each model systematically addresses a distinct stage of the cognitive-behavioral process to ensure conceptual synergy. This alignment between multi-model integration and advanced analytical workflows follows successful methodological precedents; for instance, Chen et al. integrated the Task-Technology Fit (TTF) and TAM using a hybrid analytical approach to simultaneously capture objective systemic fit and subjective user attitudes, demonstrating how complementary frameworks combined with multi-method toolkits enhance predictive accuracy and interpretive depth (Chen et al., 2026a). In our framework, the S-O-R model dictates the macro-structural sequencing, while the ISS and UTAUT dimensions operationalize the specific stimulus layer. To evaluate this integrated hierarchy without methodological redundancy, we pair linear causal path analysis with data-driven predictive modeling, ensuring that the empirical testing accurately aligns with our theoretical synthesis.

Building upon this structural logic, the present study conceptualizes the external multi-dimensional attributes of campus GenAI as environmental stimuli (S), encompassing facilitating conditions (FC), performance expectancy (PE), information quality (IQ), and service quality (SQ). These socio-technical stimuli are hypothesized to be associated with the user's internal organism (O), which is operationalized here through a parallel dual-path trust mechanism: cognitive system-like trust (AST) and affective human-like trust (AHT). Ultimately, these polarized psychological states converge to drive the behavioral response (R), manifested as students' continuance intention (CUI) toward the campus GenAI agent.

### 3.1 Facilitating conditions and trust

Within the S-O-R framework, facilitating conditions (FC) function as a theoretically relevant environmental stimulus, defined as the degree to which an individual believes that organizational and technical infrastructures exist to support system use. In the context of campus GenAI, this emphasizes access to reliable technological resources, organizational support, training and skill development, as well as user support and assistance (Rahi et al., 2019). When students perceive a robust external support system, this “structural assurance” may mitigate the inherent uncertainties of AI-generated content and reduce cognitive risks (Salimzadeh et al., 2024). Such assurance bolsters students' evaluations of the agent's professional reliability and competence, thereby potentially fostering functional system-like trust (AST). Furthermore, the relationship between facilitating conditions and AI human-like trust (AHT) is bridged through two socio-cognitive mechanisms: affective transfer and cognitive resource allocation. First, comprehensive institutional support signals organizational benevolence; through affective transfer, students project this institutional care onto the campus GenAI, implicitly viewing the agent as an empathetic extension of the university's support system. Second, facilitating conditions dissolve interactional barriers and usage anxiety, reducing the cognitive load associated with technical troubleshooting. Extending insights from human-AI collaboration literature, mitigating such operational friction can effectively alleviate users' cognitive burdens and reshape their trust dynamics (Bayer et al., 2022). Freed from operational frustrations, students experience enhanced fluidity of human-AI dialogue. This seamless engagement creates the psychological space necessary for anthropomorphic attributions (Schuetzler et al., 2020); consequently, users are more likely to perceive the AI as a social collaborator rather than a mere tool, eliciting human-like trust (AHT). Accordingly, we propose:

**H1a:** Facilitating conditions (FC) positively influence students' AI system-like trust (AST).

**H1b:** Facilitating conditions (FC) positively influence students' AI human-like trust (AHT).

### 3.2 Performance expectancy and trust

Performance expectancy (PE) denotes the degree to which an individual believes that employing GenAI will enhance their academic or research performance, serving as a foundational utility-based stimulus for trust evaluation (Venkatesh et al., 2003). In higher education, trust calibration is heavily contingent upon anticipated task outcomes: when students expect the campus agent to meaningfully improve their academic efficiency (for instance, by accelerating literature retrieval or streamlining administrative queries), this forward-looking utility appraisal reduces perceived risk and reinforces their evaluation of the system as competent and dependable, thereby hypothesized to anchor AI system-like trust (AST). Beyond the cognitive dimension, the relationship between performance expectancy and AI human-like trust (AHT) can be explained through the expectation of contextual understanding and cognitive empathy. In the context of GenAI, high performance expectancy does not merely imply mechanical speed; it encompasses the anticipation that the agent will deliver highly tailored, context-aware academic assistance. When students expect the system to accurately interpret their specific academic challenges and generate customized solutions, they implicitly attribute a level of “cognitive empathy” to the AI. This anticipation of context-aware comprehension corresponds to a perception of the agent as an insightful mentor as opposed to a passive computational tool. Consequently, this expectation of empathetic problem-solving prompts users to attribute social characteristics, such as benevolence and care, to the non-human entity, laying the psychological groundwork for evoking AI human-like trust (AHT) (Menon and Shilpa, 2023). Accordingly, we propose:

**H2a:** Performance expectancy (PE) positively influences students' AI system-like trust (AST).

**H2b:** Performance expectancy (PE) positively influences students' AI human-like trust (AHT).

### 3.3 Information quality and trust

According to the Information Systems Success (ISS) model, information quality (IQ) is a core dimension for evaluating system output, defined as the user's subjective assessment of information accuracy, completeness, and relevance (DeLone and McLean, 2003). In the context of campus GenAI, IQ serves as a critical quality stimulus driving students' cognitive evaluations. When the information provided by the agent demonstrates high precision and logical rigor, this “content reliability” effectively alleviates the cognitive burden on students when navigating academic decisions characterized by high uncertainty. Such positive perceptions of output quality directly reinforce students' recognition of the AI's expert competence and technical logic, thereby establishing robust system-like trust (AST) (Ashfaq et al., 2020).

Furthermore, the pathway from information quality to human-like trust (AHT) operates through a social-cognitive attribution mechanism. In information systems success research, technical and informational quality attributes have been empirically proven to function as foundational catalysts that activate users' deeper psychological and cognitive evaluations, rather than existing merely as static, functional outputs (Veeramootoo et al., 2018). In generative AI interactions, high information quality extends beyond factual accuracy to encompass contextually tailored and highly relevant responsiveness. When the agent consistently delivers such precise, customized academic content, this superior output caliber transitions into an interactive cue signaling “attentiveness” to students' personalized needs. This perceived attentiveness further demonstrates a form of operational benevolence, convincing users that the system is actively supporting their goals. By reducing relational uncertainty, this combination of attentiveness and benevolence encourages students to attribute supportive social traits—such as care—to the agent, thereby fostering human-like trust (AHT). Accordingly, we propose:

**H3a:** Information quality (IQ) positively influences students' AI system-like trust (AST).

**H3b:** Information quality (IQ) positively influences students' AI human-like trust (AHT).

### 3.4 Service quality and trust

Service quality (SQ) acts as an important environmental stimulus within the interaction stage, reflecting the user's overall evaluation of the support provided by the system. In the ecosystem of campus GenAI, perceived SQ is associated with a cognitive focus on the dynamic fluidity of human-AI engagement rather than static information output (Veeramootoo et al., 2018). To understand its impact on the dual-path trust mechanism, we conceptualize the interactive traits of SQ through its operational responsiveness and systematic interaction patience.

First, the functional dimension of SQ, characterized by prompt responsiveness and timely feedback, manifests operational competence and functional reliability (Roca et al., 2006). When the agent quickly and reliably resolves queries, this precise performance reduces cognitive friction and behavior-based uncertainty. This reliable technical support reinforces students' evaluations of the agent's professional credibility, serving as a driver of system-like trust (AST). Second, the interactive aspect of SQ, captured through service personalization and systematic interaction patience, fosters the psychological foundation for human-like trust. When the campus GenAI demonstrates high interaction patience—such as providing step-by-step guidance without operational fatigue—the service interaction extends beyond mere algorithmic computing to simulate supportive social engagement. Rather than embedding intrinsic emotional attributes within SQ, this superior service delivery acts as an interactive cue that prompts students to make positive social attributions. This high-quality interaction facilitates the formation of AI human-like trust (AHT) by convincing users of the agent's contextual attentiveness and relational benevolence (Gao and Waechter, 2017). Effectively, the affective attributions derived from these high-quality procedural interactions facilitate the formation of human-like trust (AHT). Accordingly, we propose:

**H4a:** Service quality (SQ) positively influences students' AI system-like trust (AST).

**H4b:** Service quality (SQ) positively influences students' AI human-like trust (AHT).

### 3.5 The dual-path trust and continuance intention

Drawing on the dual-dimensional conceptualization of trust, this study distinguishes student trust in campus GenAI into AST and AHT. Within the S-O-R framework, this dual-path mechanism serves as the pivotal psychological organism mediating the relationship between environmental stimuli and the behavioral response of continuance intention.

Specifically, AST reflects cognitive trust in the functionality, reliability, and usefulness of the agent during human-AI interactions (Califf et al., 2020). In the context of higher education, when an agent demonstrates stable technical performance and consistent academic support, students develop competency-based instrumental reliance that reduces perceived uncertainty and sustains engagement. In contrast, AHT captures the affective and relational dimension of trust, encompassing perceptions of the agent's benevolence, integrity, and social responsiveness (Balakrishnan et al., 2022). Drawing on the foundational distinction between cognition-based and affect-based trust, we conceptualize these two dimensions as complementary rather than redundant. Rather than proposing a sequential progression from cognitive to affective trust, we theorize these two dimensions as parallel processing mechanisms. Users are posited to concurrently evaluate the AI system through a cognitive-functional channel (AST) and a socio-emotional relational channel (AHT). Therefore, the present study treats AST and AHT as parallel mediating organisms within the S-O-R architecture, examining their independent contributions to CUI. These two trust dimensions collectively mitigate perceived risks and foster long-term behavioral commitment. Accordingly, we propose:

**H5a:** AI system-like trust (AST) positively influences students' continuance intention (CUI).

**H5b:** AI human-like trust (AHT) positively influences students' continuance intention (CUI).

## 4 Methodology

### 4.1 Data collection and sampling

A purposive sampling strategy was used to recruit university students from multiple higher education institutions in China that had deployed campus-wide AI agents. Data collection was conducted both online and offline. Because the target population consisted of Chinese-speaking students, the original English measurement scales were translated into Chinese and subsequently back-translated by two independent bilingual researchers to ensure semantic equivalence and cross-cultural validity. To ensure a standardized understanding of the research context and minimize cognitive bias, participants were first presented with a brief, neutral description of the campus AI agent's capabilities, accompanied by screenshots of its natural language interface. Specifically, the investigated systems primarily encompass student-facing conversational GenAI agents tailored for higher education environments, which deliver individualized academic counseling, career planning assistance, and administrative query handling via natural language interfaces.

A screening question at the beginning of the survey restricted participation to respondents who had prior experience with the agent and used it at least once a month. To capture a representative cohort of digital natives, data collection was multi-centrically conducted across higher education institutions in Shanghai that have actively implemented these institutional AI agents: Shanghai Business School and Shanghai Second Polytechnic University. Several procedural remedies were adopted to mitigate common method bias (CMB): participation was entirely voluntary and anonymous, respondents were assured that there were no correct or incorrect answers, and the order of the measurement items was counterbalanced. Given the non-invasive nature, strict anonymity, and minimal risk of the survey design, the study qualified for an ethical review exemption under regulatory guidelines for institutional and human-subject social science research. All participants provided electronic informed consent and were explicitly notified of their right to withdraw at any time without penalty.

Of the 700 responses initially collected, 633 were deemed valid after removing patterned responses and cases with missing data, yielding an effective retention rate of 90.4%. The final sample consists of full-time undergraduate students aged 17–24 (Prensky, 2001). This empirical age bracket aligns precisely with the established “digital natives” framework, characterized by individuals who have been universally exposed to information technology from early childhood. In terms of demographic composition, the sample includes 337 females (53.24%) and 296 males (46.76%). Regarding academic year distribution, the respondents comprise 160 Freshmen (25.28%), 182 Sophomores (28.75%), 176 Juniors (27.80%), and 115 Seniors (18.17%). In terms of usage frequency, 32.5% of the participants used the AI agent 1~3 times per month, whereas 45.2% engaged with the system almost daily. This interaction frequency ensures that the respondents are active users capable of providing informed evaluations.

Prior to determining the specific analytical approach, a baseline diagnostic examination was performed to evaluate the distributional characteristics of the latent constructs. As delineated in Table 1, the skewness values range from −0.803 to −0.150, and the kurtosis values range from −0.081 to 1.817, which sit within the broader conventional bounds for general structural path simulations. However, the formal univariate Shapiro-Wilk tests for all critical measurement dimensions yield test statistics with associated *p*-values strictly less than 0.001. This systematic rejection of the null hypothesis of normality heavily confirms a widespread, statistically significant deviation from a normal distribution matrix across the collected campus sample.

**Table 1:** Summary of distribution statistics for latent constructs.

Indicator	FC	PE	IQ	SQ	AST	AHT
Skewness	−0.803	−0.704	−0.486	−0.230	−0.253	−0.150
Kurtosis	1.817	1.238	0.621	−0.081	0.199	0.010
Shapiro–Wilk	0.915	0.919	0.922	0.963	0.974	0.970

### 4.2 Measurement instrument

The measurement scales used in this study were adapted from validated instruments in the fields of information systems and human-computer interaction. To ensure contextual fit, wording modifications were made to reflect the specific setting of generative AI services—such as academic counseling and career planning—within higher education environments. The final survey instrument comprised 29 items measuring seven latent constructs, all evaluated using a five-point Likert scale ranging from 1 (strongly disagree) to 5 (strongly agree).

Facilitating conditions (FC) were measured using four items adapted from Venkatesh et al. (2003) to assess the technical resources and institutional support available to students. Performance expectancy (PE) consisted of four items derived from Venkatesh et al. (2003) and Ashfaq et al. (2020), focusing on perceived efficiency gains in managing academic workloads. Information quality (IQ) was operationalized with three items based on the ISS model to evaluate the accuracy and logical consistency of agent-generated content (DeLone and McLean, 2003). Service quality (SQ) included four items adapted from DeLone and McLean (2003) and Balakrishnan et al. (2022) to capture the operational responsiveness, service personalization, and systematic interaction patience provided by the campus agent. The psychological processing mechanisms were captured through a dual-path trust framework. System-like trust (AST) was measured with six items adapted from Muir and Moray (1996) to reflect cognitive evaluations of the system's operational stability, functional competence, and data privacy. Human-like trust (AHT) utilized five items adapted from Balakrishnan et al. (2022) to measure the socio-emotional dimension of trust, centering on the perceived benevolence and social integrity of the agent. Finally, continuance intention (CUI) was measured using three items adapted from Ashfaq et al. (2020) to evaluate students' behavioral predisposition to maintain regular utilization of the campus AI agent. Before full-scale distribution, a pilot study involving 40 participants was conducted to refine the linguistic clarity of item phrasing and confirm initial scale reliability.

### 4.3 Analytical strategy

Drawing upon the theoretical framework proposed for this study, we adopt a two-stage hybrid analytical design to evaluate the hypothesized mechanisms. The analytical framework combines Partial Least Squares Structural Equation Modeling (PLS-SEM) with Random Forest (RF) classification. These two methods are complementary by design: PLS-SEM establishes theoretically grounded causal pathways to test the hypothesized mediation structure, while RF provides a data-driven validation of predictor importance that is not constrained by the linearity assumptions of SEM. This hybrid approach aligns with recent methodological shifts in educational research. As demonstrated by recent methodological benchmarks in the field (e.g., Chen et al., 2026b), analyzing complex behavioral dynamics in technology-rich environments increasingly necessitates multi-method toolkits to simultaneously capture linear causal associations and non-linear predictive patterns (Chen et al., 2026b). Together, these methods strengthen both the explanatory and predictive validity of the findings (Bao et al., 2026; Zeng et al., 2026).

#### 4.3.1 Partial least squares structural equation modeling

PLS-SEM was selected over covariance-based SEM (CB-SEM) for three reasons. First, this study is prediction-oriented and exploratory in nature, seeking to explain variance in trust and continuance intention rather than to confirm a pre-specified covariance structure; PLS-SEM is better suited to such objectives (Hair et al., 2019). Second, as indicated by the univariate diagnostics in Table 1, the manifest indicators deviate from a strict normal distribution. Because PLS-SEM utilizes a non-parametric estimation approach, it operates independently of distributed normality assumptions, making it an appropriate choice given the empirical distribution shape of the collected data (Usakli and Rasoolimanesh, 2023). Third, PLS-SEM is well-established in IS acceptance research for modeling complex mediation chains involving multiple latent constructs (Leguina, 2015), which is precisely the dual-path trust mechanism examined here. Furthermore, this causal-predictive orientation provides an empirical foundation that connects into the subsequent complementary non-parametric machine learning analysis.

The analysis proceeded in two stages. In the first stage, the measurement model was evaluated for internal reliability (Cronbach's α and composite reliability, CR), convergent validity (factor loadings and average variance extracted, AVE), and discriminant validity (Fornell–Larcker criterion). In the second stage, the structural model was estimated to test the hypothesized paths. Statistical significance was assessed via bootstrap resampling with 5,000 iterations, which generates empirical *t*-statistics and *p*-values without requiring distributional assumptions (Zhong and Maccalla, 2024). Effect sizes were quantified using Cohen's *f*^2^, and the model's predictive relevance was evaluated using Stone-Geisser's *Q*^2^. All PLS-SEM analyses were conducted using the plspm package (v0.5.1) in Python 3.12.

#### 4.3.2 Random forest classification

To capture potential interaction effects that PLS-SEM cannot detect, a Random Forest (RF) classifier was trained to predict students' continuance usage intention. RF is an ensemble learning method that constructs a large number of decision trees on bootstrapped subsamples of the data and aggregates their predictions, yielding robust estimates that are resistant to overfitting (Breiman, 2001). Its key advantage in the present context is that it makes no assumptions about the functional form of predictor-outcome relationships, thereby serving as a model-agnostic complement to the linear SEM framework.

In our valid survey sample, CUI was dichotomized using the sample median score of 4 as the operational threshold. Substantively, a score strictly greater than four serves as a meaningful indicator of full acceptance and strong embrace of the technology, whereas scores ≤ 4 represent neutrality or hesitation. This threshold segregates the dataset into 416 cases in the “Hesitant” class (*CUI* ≤ 4, accounting for 65.7% of the total sample) and 217 cases in the “Active” (*CUI*>4, accounting for 34.3% of the total sample).

The six construct-level latent variable scores (FC, PE, IQ, SQ, AST, and AHT) served as predictors, with latent variable scores computed as the item mean for each construct and priorly standardized to align features. The dataset was partitioned into a training set (80%, *N* = 506) and a held-out test set (20%, *N* = 127). To ensure the stability of feature estimates and strictly adhere to predictive reporting norms, a randomized search with five-fold cross-validation (*RandomizedSearchCV*) was implemented on the training set over 100 iterative combinations. The hyperparameter optimization framework expanded across dynamic parameter spaces, tuning key configurations including the total number of ensemble trees (n_estimators sampled uniformly from 100 to 800), maximum tree growth layers (max_depth ranging from 5 to 30 alongside unconstrained growth), minimum structural sample splits (min_samples_split from 2 to 20), minimum leaf node constraints (min_samples_leaf from 1 to 10), split randomizations (max_features), and adjusting for minor distribution asymmetries via class_weight.

The optimized Random Forest classifier was then validated on the held-out test set using overall accuracy, precision, recall, and F1-score. Baseline feature importance was quantified via mean decrease in impurity (MDI) across the optimized ensemble to yield a stabilized ranking of relative predictor priorities (Breiman, 2001). Additionally, to formally unpack the potential non-linear threshold effects and uncover complex interaction boundaries that traditional linear structural equation models inherently obscure, a model-agnostic *post-hoc* verification was executed by generating a one-dimensional Partial Dependence Plot (PDP) specifically tracking the localized marginal behavior of PE across the predicted distribution.

#### 4.3.3 Rationale for the hybrid approach

The two methods address distinct but complementary research objectives. PLS-SEM tests whether the theoretically derived causal pathways—specifically, whether dual-path trust mediates the stimulus-response relationship—are statistically supported, and provides standardized path coefficients for hypothesis evaluation. RF then independently ranks all predictors by their contribution to CUI prediction, offering a data-driven check on the SEM findings and revealing whether any construct carries predictive weight through non-linear mechanisms that the linear model cannot capture. This hybrid design responds to calls in the IS literature for methodological pluralism in technology acceptance research and ensures that the conclusions are robust across both theory-confirmatory and data-driven analytical lenses (Zeng et al., 2026).

## 5 Results

### 5.1 Measurement model assessment

The measurement model was evaluated using Partial Least Squares Structural Equation Modeling (PLS-SEM), assessing internal reliability, convergent validity, and discriminant validity prior to structural model testing.

#### 5.1.1 Internal reliability and convergent validity

As shown in Table 2, all Cronbach's α values ranged from 0.896 to 0.932, exceeding the recommended threshold of 0.70. Composite reliability (CR) values ranged from 0.924 to 0.952, all well-above the 0.70 threshold (Hair et al., 2019). All indicator factor loadings exceeded 0.70 (range: 0.786~0.931), and average variance extracted (AVE) values ranged from 0.694 to 0.846, all surpassing the 0.50 threshold (Fornell and Larcker, 1981). These results confirm satisfactory internal reliability and convergent validity for all seven constructs.

**Table 2:** Reliability, convergent validity, and factor loadings.

Construct	Item	Loading	Cronbach's α	CR	AVE
Facilitating Conditions (FC)	A1	0.867	0.899	0.930	0.769
	A2	0.892			
	A3	0.867			
	A4	0.882			
Performance Expectancy (PE)	A5	0.906	0.929	0.949	0.824
	A6	0.907			
	A7	0.906			
	A8	0.912			
Information Quality (IQ)	A9	0.914	0.909	0.943	0.846
	A10	0.915			
	A11	0.929			
Service Quality (SQ)	A12	0.903	0.932	0.952	0.832
	A13	0.901			
	A14	0.931			
	A15	0.912			
AI System-like Trust (AST)	B1	0.848	0.910	0.931	0.694
	B2	0.850			
	B3	0.792			
	B4	0.848			
	B5	0.786			
	B6	0.870			
AI Human-like Trust (AHT)	B7	0.823	0.896	0.924	0.707
	B8	0.819			
	B9	0.851			
	B10	0.847			
	B11	0.865			
Continuance Usage Intention (CUI)	C1	0.898	0.902	0.939	0.836
	C2	0.925			
	C3	0.920			

#### 5.1.2 Discriminant validity

Discriminant validity was assessed using the Fornell–Larcker criterion: the square root of each construct's AVE (bold diagonal) must exceed its Pearson correlations with all other constructs (lower triangle), where correlations are computed from construct-level latent variable scores. As shown in Table 3, all diagonal values exceed the corresponding off-diagonal correlations in their respective rows and columns, confirming adequate discriminant validity across all construct pairs. The highest inter-construct correlation observed is between AST and AHT (*r* = 0.788), which remains below AST's $\sqrt{AVE}\;$of 0.833 and AHT's $\sqrt{AVE}\;$of 0.841, indicating that the two trust dimensions, while related, are empirically distinct. The stimulus constructs IQ and SQ exhibit the strongest inter-stimulus correlation (*r* = 0.731), yet both diagonal values (0.920 and 0.912) comfortably exceed this value. To provide a more granular evaluation of construct distinctness, the Heterotrait–Monotrait (HTMT) ratio was additionally computed (Henseler et al., 2015). The AST–AHT pair yielded an HTMT value of 0.874. While this value marginally exceeds the conservative 0.850 threshold, it remains well within the 0.900 limit recommended for complex models in human-AI interaction research, where cognitive and affective mechanisms exhibit theoretical interdependence. This result, combined with the Fornell–Larcker findings, provides a multi-faceted verification of the model's measurement properties.

**Table 3:** Discriminant validity—Fornell–Larcker criterion.

Item	FC	PE	IQ	SQ	AST	AHT	CUI
FC	0.877						
PE	0.512	0.908					
IQ	0.487	0.693	0.920				
SQ	0.503	0.621	0.731	0.912			
AST	0.501	0.614	0.724	0.685	0.833		
AHT	0.472	0.595	0.691	0.662	0.788	0.841	
CUI	0.432	0.556	0.642	0.629	0.654	0.679	0.914

To further substantiate this discriminant validity profile, a comprehensive manifest indicator cross-loadings examination was conducted to verify indicator-to-construct alignment, with the complete metric documented in Table 4. The empirical matrix confirms that all 11 items assigned to the trust pathways load higher on their designated latent variable than on the alternative trust construct. Specifically, the AST indicators (B1 to B6) exhibit primary factor loadings ranging from 0.786 to 0.870, exceeding their cross-loadings on AHT, which range from 0.683 to 0.781. Similarly, the AHT indicators (B7 to B11) exhibit primary loadings between 0.819 and 0.865, while their cross-loadings on AST remain restricted between 0.695 and 0.751. This pattern indicates that the manifest items isolate their respective latent dimensions without structural cross-contamination.

**Table 4:** Manifest indicator cross-loadings matrix for trust constructs.

Item	FC	PE	IQ	SQ	AST	AHT	CUI	Group
B1	0.395	0.436	0.578	0.480	0.848	0.683	0.528	
B2	0.467	0.553	0.651	0.556	0.850	0.742	0.618	
B3	0.490	0.549	0.664	0.495	0.792	0.700	0.617	AST
B4	0.387	0.420	0.536	0.475	0.848	0.696	0.465	
B5	0.337	0.340	0.455	0.418	0.786	0.697	0.440	
B6	0.467	0.528	0.645	0.480	0.870	0.781	0.591	
B7	0.374	0.448	0.510	0.576	0.714	0.823	0.528	
B8	0.455	0.471	0.603	0.456	0.751	0.819	0.561	
B9	0.401	0.467	0.529	0.585	0.695	0.851	0.554	AHT
B10	0.424	0.440	0.573	0.458	0.742	0.847	0.574	
B11	0.484	0.567	0.630	0.538	0.726	0.865	0.628	

### 5.2 Structural model assessment

#### 5.2.1 Predictive relevance

The structural model's explanatory power was evaluated using the coefficient of determination (*R*^2^) and Stone-Geisser's *Q*^2^ predictive relevance. As reported in Table 5, the model explains 53.6% of the variance in AI System-like Trust (*R*^2^ = 0.536), 52.7% in AI Human-like Trust (*R*^2^ = 0.527), and 47.7% in Continuance Usage Intention (*R*^2^ = 0.477). All *R*^2^ values exceed the 0.26 threshold for substantial explanatory power (Cohen, 2013), and all *Q*^2^ values are positive, confirming the model's predictive relevance.

**Table 5:** Predictive relevance of endogenous constructs.

Construct	*R* ^2^	*Q* ^2^
AST	0.536	0.372
AHT	0.527	0.373
CUI	0.477	0.399

#### 5.2.2 Hypothesis testing

**Figure 1:**
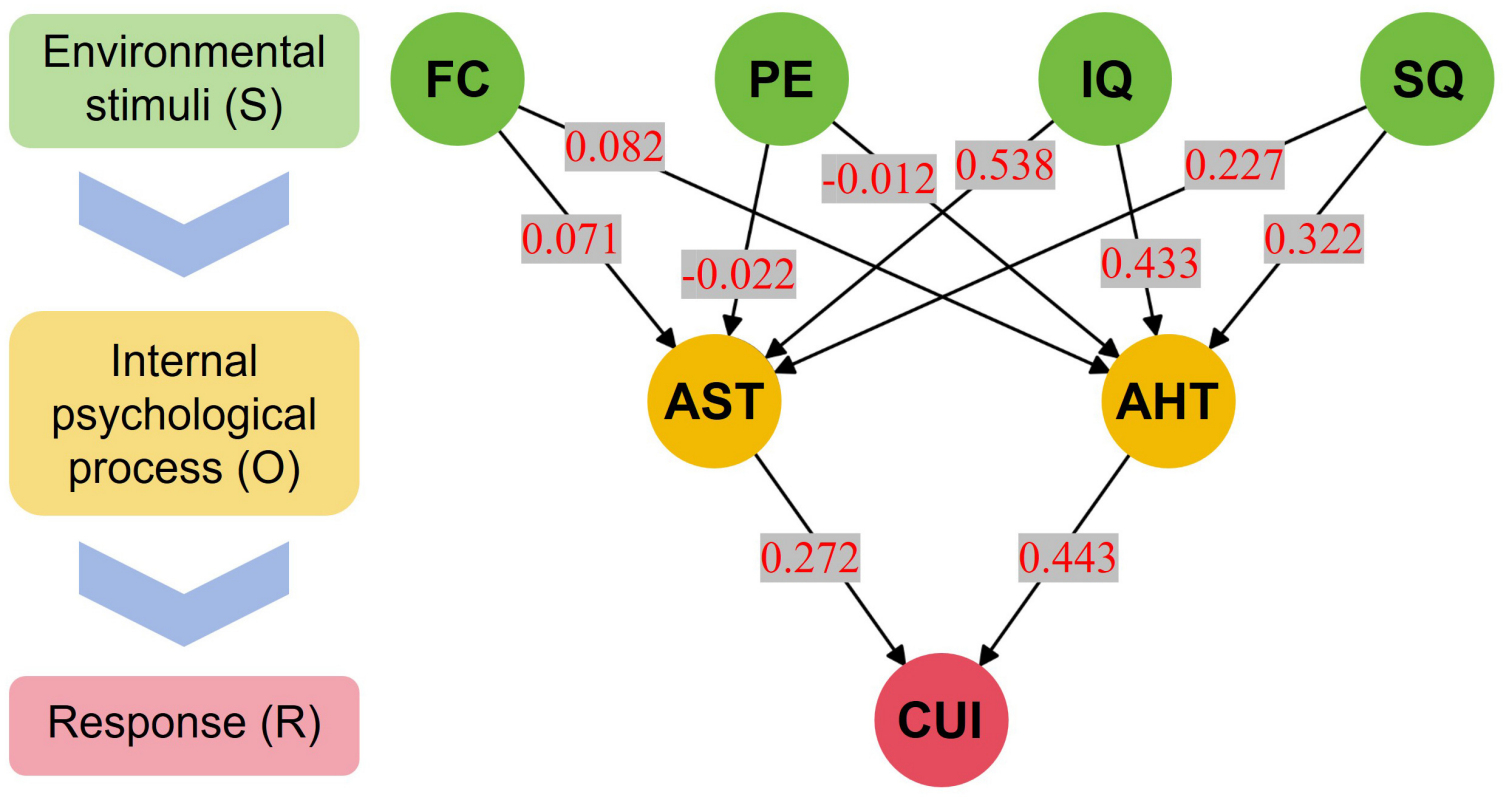
Structural model results with standardized path coefficients.

Structural paths were estimated using PLS-SEM with bootstrap resampling (5,000 iterations) to derive *t*-statistics and assess significance. Standardized path coefficients (β), effect sizes (*f* ^2^), and hypothesis outcomes are presented in Table 6. Effect size *f* ^2^ is interpreted as negligible (< 0.02), small (0.02–0.15), medium (0.15–0.35), or large (>0.35). The results reveal a clear pattern of differential stimulus effects. Information Quality (IQ) emerged as the dominant predictor of both trust dimensions, exerting a positive effect on AST (β = 0.538, *p* < 0.001) and a substantial effect on AHT (β = 0.433, *p* < 0.001), supporting H3a and H3b. Service Quality (SQ) also significantly predicted both AST (β = 0.227, *p* < 0.001) and AHT (β = 0.322, *p* < 0.001), supporting H4a and H4b. In contrast, Facilitating Conditions (FC) and Performance Expectancy (PE) exerted negligible and non-significant effects on both trust dimensions (FC → AST: β = 0.071; FC → AHT: β = 0.082; PE → AST: β = −0.022; PE → AHT: β = −0.012; all *p* > 0.05), leading to the rejection of H1a, H1b, H2a, and H2b. Regarding the organism-to-response pathways, both trust dimensions significantly predicted Continuance Usage Intention. AI Human-like Trust exerted a stronger effect on CUI (β = 0.443, *p* < 0.001) than AI System-like Trust (β = 0.272, *p* < 0.01), a formal bootstrap difference test was executed to evaluate this variation. The 95% percentile bootstrapped confidence interval for their direct path difference (Δβ = 0.171) was [−0.032, 0.360]. Since this interval contains zero, the direct paths from AST and AHT to CUI are statistically equivalent at the 5% significance level, indicating that their linear confidence intervals overlap. This statistical equivalence indicates that the null hypothesis stating that these two trust dimensions exert equal predictive effects on Continuance Intention cannot be statistically rejected. This non-significance implies that both technology-centric and human-centric trust pathways remain parallel drivers of student commitment, though their cross-sectional equivalence requires cautious longitudinal validation. Figure 1 displays all estimated paths with standardized coefficients; *R*^2^ values are reported beneath each endogenous construct; path coefficients are derived from bootstrap resampling (5,000 iterations).

**Table 6:** Structural model results and hypothesis testing.

Hypothesis	Path	β	*p*-value	*f* ^2^	Decision
H1a	FC → AST	0.071	>0.05	0.007	Not supported
H1b	FC → AHT	0.082	>0.05	0.009	Not supported
H2a	PE → AST	−0.022	>0.05	0.001	Not supported
H2b	PE → AHT	−0.012	>0.05	0.000	Not supported
H3a	IQ → AST	0.538	< 0.001	0.289	Supported
H3b	IQ → AHT	0.433	< 0.001	0.188	Supported
H4a	SQ → AST	0.227	< 0.001	0.052	Supported
H4b	SQ → AHT	0.322	< 0.001	0.104	Supported
H5a	AST → CUI	0.272	< 0.01	0.074	Supported
H5b	AHT → CUI	0.443	< 0.001	0.196	Supported

#### 5.2.3 *Post-hoc* diagnostic inspection

To evaluate the empirical necessity of preserving the dual-path trust framework (AST and AHT) within the structural network, a nested model fit comparison was executed by estimating an alternative structural configuration where the AST and AHT dimensions were collapsed into a single, unified trust dimension. The empirical comparison validates the retention of the hypothesized framework over the collapsed alternative. The hypothesized dual-factor framework explains a higher proportion of variance in the primary downstream target construct, yielding an *R*^2^ for CUI of 0.477, compared to 0.423 in the restricted single-construct model. Furthermore, the dual-factor architecture demonstrates superior global exploratory fit, achieving a higher Goodness-of-Fit (GoF) index of 0.630 compared to 0.578 for the merged model, alongside an increased average trust-related AVE profile (0.700 vs. 0.652). These collective assessments provide empirical justification for treating AST and AHT as distinct parallel predictive pathways within the structural model evaluation.

Concurrently, inner model collinearity diagnostics were evaluated based on latent variable scores. For the antecedents predicting AST and AHT, the inner Variance Inflation Factors (VIF) range from 1.859 to 3.106 (PE = 3.106). For the pathways predicting CUI, the inner VIF values for AST and AHT both stand at 3.269. These vectors sit within the threshold of 5.000, confirming the absence of critical multicollinearity (Hair et al., 2017). Furthermore, a *post-hoc* auxiliary regression reveals that 58.98% of the variance in PE is explained concurrently by IQ and SQ. When these quality attributes are isolated via nested comparisons, the localized indirect linkages of FC and PE onto the trust domains are significant, whereas their paths are attenuated under global simultaneous estimation.

### 5.3 Mediation analysis

To examine the dual-path trust mechanism, we assessed the indirect effects of the four stimuli on CUI through AST and AHT separately. Indirect effects were computed as the product of the two constituent path coefficients such as the path from IQ to AST multiplied by the path from AST to CUI, and significance was evaluated using 95% bias-corrected bootstrap confidence intervals with 5,000 replications. A confidence interval that does not include zero indicates statistical significance. The results, presented in Table 7, reveal that IQ exerts the strongest total indirect effect on CUI (β = 0.338), operating through both the AST pathway (β = 0.146) and the AHT pathway (β = 0.192). SQ also demonstrates a substantial total indirect effect (β = 0.205), with the AHT-mediated path (β = 0.143) exceeding the AST-mediated path (β = 0.062). These findings confirm that both trust dimensions function as significant mediators between content and service quality stimuli and sustained usage commitment. In contrast, the indirect effects of FC (total β = 0.055) and PE (total β = −0.011) on CUI through both trust pathways are statistically non-significant, consistent with the non-significant direct paths from these stimuli to the trust constructs. Hierarchical nesting diagnostics indicate that the non-significance of these pathways is an empirical function of variance mediation, wherein the predictive variance of FC and PE is channeled through the proximate IQ and SQ constructs within the integrated structural system.

**Table 7:** Mediation analysis—indirect effects on CUI via AST and AHT.

Path	Via AST (β)	Via AHT (β)	Total indirect (β)
IQ → [AST/AHT] → CUI	0.146	0.192	0.338
SQ → [AST/AHT] → CUI	0.062	0.143	0.205
FC → [AST/AHT] → CUI	0.019	0.036	0.055
PE → [AST/AHT] → CUI	−0.006	−0.005	−0.011

### 5.4 Random forest analysis

To complement the PLS-SEM findings and capture potential non-linear relationships, a Random Forest classifier was trained to predict students' Continuance Usage Intention. CUI was dichotomized at a threshold of four (on the five-point scale) into “Hesitant” (CUI ≤ 4) and “Active” (CUI > 4) categories. The six construct-level latent variable scores (FC, PE, IQ, SQ, AST, and AHT) served as predictors. The model was trained on 80% of the sample (*n* = 506) and evaluated on the remaining 20% (*n* = 127), with feature importance derived from mean decrease in impurity across 500 trees.

#### 5.4.1 Model performance

The optimized Random Forest classifier achieved an overall accuracy of 87.4% on the held-out test set. As shown in Table 8, the model demonstrated particularly strong and stable predictive capability for the “Hesitant” class (precision = 0.89, recall = 0.95, and F1-score = 0.92), while capturing robust boundaries for the “Active” class (precision = 0.81, recall = 0.66, and F1-score = 0.72). The weighted average F1-score of 0.87 confirms high overall predictive validity. Furthermore, to control for potential sample-specification artifacts arising from a single data split, a complementary five-fold cross-validation protocol was executed on the classification matrix. The iterative cross-validation routine yielded a stable mean classification accuracy of 0.83 ± 0.03. This consistent distribution demonstrates that the predictive generalizability of the environmental stimuli and trust organism framework remains invariant across distinct validation folds and does not represent an artifact of localized overfitting.

**Table 8:** Random forest classification performance.

Class	Precision	Recall	F1-score
Hesitant (CUI ≤ 4)	0.89	0.95	0.92
Active (CUI > 4)	0.81	0.66	0.72
Weighted average	0.87	0.87	0.87

#### 5.4.2 Feature importance and convergence with PLS-SEM

Feature importance scores and their correspondence with PLS-SEM results are presented in Table 9. AHT emerged as the single most dominant predictor of CUI, capturing a substantial relative weight of 0.5094 (50.94% of total importance), followed by IQ and AST. Notably, the cumulative feature importance of the top three predictors reached 84.21%, indicating that the students' decision boundaries are heavily dominated by affective trust components and information quality. Conversely, PE, SQ, and FC reside within the lower tier of predictive priority. The RF importance rankings converge with the PLS-SEM results in three key respects. First, both methods identify AHT as the primary determinant of CUI, with the prominent RF importance score corroborating the larger PLS-SEM path coefficient for AHT → CUI (β = 0.443) relative to AST → CUI (β = 0.272). Second, IQ ranks as the most critical environmental stimulus variable in both analyses, consistent with its dominant structural paths in the linear model. Third, FC ranks last among all predictors in the RF analysis, which aligns seamlessly with its non-significant path in the PLS-SEM framework, confirming its marginal incremental utility.

**Table 9:** Random forest feature importance and comparison with PLS-SEM results.

Rank	Construct	RF importance	Cumulative %	PLS-SEM path to CUI
1	AHT (AI Human-like Trust)	0.5094	50.94%	β = 0.443
2	IQ (Information Quality)	0.1682	67.76%	β = 0.338
3	AST (AI System-like Trust)	0.1645	84.21%	β = 0.272
4	PE (Performance Expectancy)	0.0751	91.72%	β = −0.011
5	SQ (Service Quality)	0.0532	97.05%	β = 0.205
6	FC (Facilitating Conditions)	0.0295	100.0%	β = 0.055

**Figure 2:**
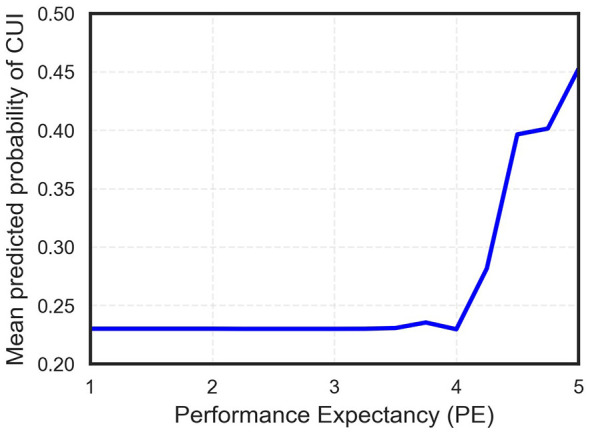
Partial dependence plot of PE on high CUI.

A notable divergence, however, is observed regarding Performance Expectancy (PE). Although PE yielded a non-significant linear total indirect path in PLS-SEM (β = −0.011), it retains a meaningful predictive footprint in the Random Forest ensemble (0.0751), outranking SQ and FC. Rather than an analytical mismatch, this divergence is elucidated by the *post-hoc* Partial Dependence Plot (Figure 2), which uncovers a distinct non-linear threshold activation mechanism. While the driving force of PE remains completely dormant across the lower-to-average spectrum (explaining its diluted non-significance in the linear SEM framework), it triggers a sharp, localized surge in the probability of high CUI once the premium experiential threshold of 4.0 is crossed. These findings imply that enhanced interface usability tends to operate via a step-function dynamic rather than a continuous progression. Consequently, traditional linear structural equation models may exhibit limited sensitivity in detecting such localized inflection points within the user decision matrix.

## 6 Discussion

### 6.1 The epistemic and relational foundations of dual-path trust

The empirical results reveal a nuanced divergence in how environmental stimuli are associated with the dual-path trust organism. Information Quality (IQ) emerged as the dominant anchor for both AST (β = 0.538) and AHT (β = 0.433), while Service Quality (SQ) demonstrated a stronger affinity with AHT (β = 0.322) than with AST (β = 0.227). While the impact of IQ on AST aligns with prior literature asserting that content accuracy and logical rigor serve as critical signals of an AI system's technical competence (Zhang et al., 2025; Ashfaq et al., 2020), its powerful activation of AHT (β = 0.433) provides a deeper socio-cognitive insight. Within the campus GenAI ecosystem, structural accuracy and logical coherence transcend raw functional utility; they function as advanced anthropomorphic cues. Under the Computers are Social Actors (CASA) paradigm, when an agent delivers highly nuanced, intellectually rigorous counseling, students tend to attribute a form of “cognitive wisdom” and benevolence to the artifact, thereby satisfying relational expectations and seeding affective trust (Balakrishnan et al., 2022). Conversely, the comparative dominance of SQ over IQ in driving AHT underlines the agent's potential transition from a functional tool to an interactive relational partner. Because SQ captures behavioral responsiveness and interactional empathy, it directly stimulates social-emotional attributions rather than mere cognitive-functional evaluations (Balakrishnan et al., 2022; Kim et al., 2025). Personalized, empathetic feedback generates a perceptible sense of social presence, mitigating the inherent anxiety of deep learning architectures and transforming mechanical encounters into reassuring social support. This dynamic extends the application of parallel trust-risk frameworks in educational AI by demonstrating that service interactivity is a more potent activator of affective trust than of cognitive trust in the campus context (Xia et al., 2026). It is worth noting that while the empirical overlap between AST and AHT reflects a high degree of statistical association (HTMT = 0.874), this should be interpreted as a manifestation of psychological synergy rather than measurement redundancy (Lankton et al., 2015). Given the theoretical interdependence of cognitive and affective trust in AI-mediated environments, these dimensions operate as reinforcing, parallel pathways that collectively sustain student engagement, rather than as isolated psychological constructs.

### 6.2 Functional baseline constraints and holistic evaluation

The empirical matrix reveals that the influence of PE and FC is absorbed when estimated concurrently with systemic quality attributes. Rather than indicating that functional utility is absent, this pattern reflects a holistic evaluation mechanism characteristic of digital natives, who perceive campus generative AI tools as integrated educational ecosystems. Methodologically, this systemic integration is captured by the inner variance inflation properties (Hair et al., 2019), where PE exhibits an inner Variance Inflation Factors (VIF) of 3.106, driven by its high overlapping variance with IQ and SQ, which together account for 59.0% of its total variance (*R*^2^ = 0.590). Within this parallel architecture, Performance Expectancy (PE) operates not as an isolated linear antecedent, but as a baseline performance constraint that is conceptually subsumed under the immediate evaluative criteria of epistemic rigor (IQ) and responsive engagement (SQ). Consequently, the additive structural path coefficients do not exhibit independent linear significance for PE, as its predictive variance is systematically channeled through the proximate quality pathways.

This statistical pattern clarifies the functional hygiene dynamics of generative AI within the higher education continuum. Rather than indicating that functional performance is irrelevant to students, the overlap of variance suggests that basic task acceleration and instrumental accessibility have mutated into baseline hygiene assumptions. Within Herzberg's dual-factor framework, hygiene factors are indispensable structural requirements whose presence avoids dissatisfaction but does not linearly drive positive motivation or continuous behavioral commitment (Herzberg et al., 1960). For connected learners, conversational availability and fundamental performance stability operate as pre-configured infrastructure. Incremental linear gains in processing speed or functional interface access do not translate into a proportional, constant increase in psychological trust or continuous usage intention. This interpretation aligns with the alternative non-parametric machine learning analysis, where PE re-emerges with a distinct predictive footprint. As further substantiated via exploratory *post-hoc* inspections, PE operates through a non-linear threshold dynamic rather than a continuous progression, acting as a conditional threshold catalyst that functions primarily within the positive spectrum of the measurement scale. Such an empirical variance demonstrates that performance expectancy continues to operate as an essential baseline threshold undergirding the entire human-AI interactive matrix.

### 6.3 Parallel trust pathways and discrepancy assessment

The mediation analysis confirms that both AST and AHT function as significant mediators between the quality stimuli and CUI, supporting the dual-path trust mechanism proposed in the S-O-R framework. The total indirect effect of IQ on CUI (β = 0.338) exceeds that of SQ (β = 0.205), indicating that information quality represents a primary stimulus for activating user trust. Critically, the formal bootstrap difference test reveals that the direct paths of AHT and AST to CUI are statistically equivalent (Δβ = 0.171, 95*%CI* = [−0.032, 0.360]), indicating that the null hypothesis of equal predictive effects cannot be statistically rejected. This non-significance suggests that both technology-centric and human-centric trust pathways remain parallel drivers of student commitment within the current empirical boundary, though their cross-sectional equivalence requires cautious longitudinal validation rather than an absolute assumption of mathematical symmetry.

To understand the structural properties of these parallel mechanisms, the two paths can be conceptualized through a layered functional complementarity (Li et al., 2024). Cognitive trust (AST) reflects an evaluation grounded in performance track-records, algorithmic predictability, and data privacy safeguards, representing cognitive alignment as a baseline structural dimension of user compliance. If the system lacks this technical competence framework, user engagement exhibits vulnerability, signaling that cognitive alignment serves as a core infrastructure. In a generative AI context, instances of sporadic algorithmic hallucinations or network disruptions relate directly to the recalibration of this baseline alignment. Concurrently, Affective Trust (AHT) encompasses perceived benevolence, shared social alignment, and relational reciprocity. Rather than operating sequentially, affective trust functions concurrently as a complementary relational anchor that underpins user retention under conditions of continuous interaction.

In the campus AI interface, this affective dimension relates to a tolerance for occasional technical limitations or minor errors during human-AI interactions. The bootstrap difference testing further clarifies this pattern by uncovering a localized operational boundary within the mediation flows. While the indirect effects of IQ via the two channels are statistically equivalent, a divergence occurs for SQ. The indirect effect of SQ on CUI via the AHT channel (mean = 0.142) exceeds its counterpart via the AST channel (mean = 0.062), yielding a bootstrap difference interval above zero. This specialized divergence demonstrates that while cognitive attributes relating to the structural threshold of the system, conversational and responsive service attributes preferentially involve the affective trust channel to translate service experiences into continuous usage intentions.

### 6.4 Cross-method validation: reconciling structural and machine learning divergences

The ensemble machine learning analysis via an optimized Random Forest (RF) classifier provides a model-agnostic validation of the linear PLS-SEM findings while uncovering a structural variance in variable prioritization. Methodological convergence is achieved in three distinct respects: both methodologies identify AHT as a primary driver of CUI, position IQ as a critical environmental stimulus, and relegate FC to the lowest tier of importance. This multi-method alignment supports the structural robustness of the proposed dual-path trust framework against model-specification artifacts. However, a granular divergence is observed regarding the relative positioning of the trust dimensions. While the linear estimation in PLS-SEM indicates that both AHT and AST exert concurrently significant and substantial linear driving forces within a globally aggregated structural model, the data-driven ensemble learning analysis clarifies that under recursive partitioning, AHT commands a substantially higher conditional split importance relative to AST. Furthermore, the optimized RF model achieved a robust overall classification accuracy of 0.874 on the held-out test set, heavily corroborating the empirical predictive validity of the synthesized variable framework.

Additionally, an empirical divergence emerges regarding PE. Although PE yielded a non-significant linear path within the PLS-SEM framework (β = −0.01), it retains a meaningful predictive footprint within the non-parametric RF classifier, securing an importance score of 0.0751 (ranking 4th) and outperforming SQ and FC. Rather than an analytical contradiction, this variance reflects the alternative operational boundaries of pragmatic utility captured by different statistical estimators within the human-AI interaction continuum. The statistical non-significance in PLS-SEM implies that within a globally aggregated linear model, incremental efficiency gains do long-term commitment no proportional, constant pressure. Conversely, the predictive signal captured by the RF ensemble suggests that PE operates through a non-linear threshold activation dynamic that traditional additive linear equations are less equipped to identify (Zeng et al., 2026).

This localized step-function behavior is explicitly unpacked via the *post-hoc* non-linear diagnostic inspection. The empirical curve indicates that the marginal utility of PE remains entirely dormant across the lower-to-average spectrum, explaining its diluted non-significance under the global linearity assumption of SEM. However, a sharp non-linear inflection occurs once user perception clears a critical threshold toward the positive spectrum of the measurement scale. This trend implies that while basic task acceleration operates as a functional hygiene factor for connected learners, a premium threshold of computational competence tends to function as a conditional threshold catalyst within the user decision matrix. If a campus AI agent's efficiency is evaluated to be below this critical functional threshold, the associated user behavior is characterized by avoidance, irrespective of the agent's relational attributes. Consequently, while relational and epistemic qualities, specifically AHT and IQ, act as the active engines driving post-adoption continuance, functional utility (PE) constitutes a baseline operational boundary and a non-linear trigger zone within which these social dynamics unfold (Bao et al., 2026; Zeng et al., 2026).

### 6.5 Limitations and future research

Several methodological limitations must be acknowledged. First, the cross-sectional design captures only a static snapshot of user perceptions rather than the long-term developmental trajectory of trust. This reliance introduces cross-sectional bias, as static statistical associations cannot empirically isolate the temporal sequencing of cognitive and affective trust adjustments. Second, the use of a convenience, non-probability sampling strategy restricted to specific higher education institutions limits the geographical and institutional generalizability of the empirical matrix. These dynamics remain bounded by localized campus configurations, cultural expectations, and institutional policies. Third, beyond these design constraints, specific statistical and measurement defects inherent in our quantitative approach must be explicitly recognized as limitations. Regarding structural modeling, the PLS-SEM path analysis is fundamentally restricted by a global linearity assumption across aggregated distributions. This mathematical boundary compromises the model's sensitivity in detecting localized, non-linear step-functions or conditional inflection points within the user decision matrix. Fourth, regarding predictive estimation, the adopted inconsistent variable coding strategy introduces a distinct measurement defect. While the artificial median-dichotomization used to split the continuance intention variable was operationalized to isolate discrete boundaries between active technological embrace and hesitation, this procedure inevitably discards a portion of the continuous structural variance and obscures more subtle gradations within behavioral commitment profiles. Finally, due to the exclusive reliance on single-source, self-reported subjective survey indicators, the measurement properties remain vulnerable to common method bias. This structural reliance prevents the concurrent integration of objective, real-time automated behavioral logging metrics to cross-verify psychological perceptions.

Future research should therefore leverage longitudinal frameworks and continuous regression-based machine learning, coupled with objective automated behavioral logging, to transition from static association to a real-time assessment of user behavior. Future studies should formally operationalize and statistically test for potential non-linear relationships and threshold verification, explicitly examining whether utilitarian affordances exhibit a distinct saturation effect as user experience accumulates. Additionally, future efforts should aim to expand the empirical scope by incorporating larger and more diverse datasets, including cohorts from multiple Chinese universities and international institutions, to validate whether the observed trust-mediated mechanisms remain consistent across varied educational ecosystems and cross-cultural contexts (Chen J. et al., 2025). Crucially, future replication efforts should explore alternative data-driven frameworks, such as unsupervised clustering or automated threshold optimization, to capture the natural partitions within user behavioral data. This approach would successfully reduce the reliance on domain-specific prior knowledge while further refining the threshold effects identified in this preliminary classification.

## 7 Conclusions

This study examines the associations between the environmental configurations of campus GenAI tools and the psychological states of user trust to predict post-adoption commitment. Analyzing this quality-trust-continuance nexus yields two primary theoretical implications for human-AI interaction in higher education. First, within the examined educational context, Information Quality (IQ) and Service Quality (SQ) serve as critical structural anchors for dual-path trust. Conversely, Facilitating Conditions (FC) and Performance Expectancy (PE) exhibit non-significant direct linear paths within the globally aggregated structural model. This divergence provides a conceptual parallel to dual-factor frameworks, suggesting that functional utility may operate under distinct parameter boundaries once baseline operational expectations are satisfied. Rather than implying functional irrelevance, this structural pattern, when cross-validated with non-parametric machine learning insights, underscores that PE tends to operate via a conditional threshold dynamic, transitioning into an active catalyst primarily within the positive spectrum of user perception. Second, affective relational attachment (AI Human-like Trust) serves as a principal psychological anchor associated with behavioral commitment alongside cognitive-functional reliance. While cognitive evaluations establish the rational foundation, the perception of relational benevolence—triggered as an attributional response to superior service delivery—is linked with relational loyalty. This psychological dimension is positively associated with students' continuance intentions, demonstrating robust invariance across distinct algorithmic performance spectrums. Practically, these insights suggest that institutional developers and educators should prioritize continuous content verification and the programmatic implementation of responsive, empathetic interaction methods over mere functional scaling to cultivate sustained, long-term user engagement with campus GenAI systems.

## Data Availability

The original contributions presented in the study are included in the article/supplementary material, further inquiries can be directed to the corresponding author.
